# How the COVID-19 Pandemic Impacted the Perception of Climate Change in the UK

**DOI:** 10.1177/00027642221085885

**Published:** 2022-04-24

**Authors:** Gabriele Ruiu, Maria Laura Ruiu, Massimo Ragnedda

**Affiliations:** 1 9312 University of Sassari, Sassari, Italy; 2 Department of Social Sciences, 5995 Northumbria University, Newcastle Upon Tyne, UK

**Keywords:** climate change, COVID-19, environmental awareness, pro-environmental behaviour

## Abstract

The COVID-19 pandemic erupted during the climate change (CC) crisis, forcing individuals to adapt abruptly to a new scenario, and triggering changes in everyone’s lifestyles. Based on a sample of the UK population (*N* = 1013), this paper investigates how the COVID-19 pandemic invited/forced individuals to reflect upon a more sustainable way of life (which might be enhanced by the use of digital technologies for daily activities) and to (re)consider the anthropogenic impact on the environment. The results show that older individuals tend to be less sceptic around the human impact on CC. Other control variables such as income, gender and employment status have a limited impact on this attitude towards CC. Secondly, the findings indicate a clear separation between those with a minimal level of education, who support the natural origin of CC, while individuals with a higher level of education believe that CC is caused by human actions. Finally, on average, younger and more educated individuals tend to associate the COVID-19 pandemic with an opportunity to promote an eco-friendly world and to adopt an eco-sustainable approach.

## Introduction

The COVID-19 pandemic showed the intertwined connections between natural and societal systems, by highlighting that biodiversity loss and intensive food systems increase the probability of zoonotic diseases. More specifically, the COVID-19 pandemic occurred in parallel to the climate change (CC) crisis, and was responsible for unintended, short-term, positive consequences on environmental systems due to, for example, decreased pollution from industries and vehicle emissions (European Environment Agency, 2020). This has been possible thanks to the restrictive measures on people’s movement. Therefore, the COVID-19 crisis has forced individuals to suddenly adapt to the new situation, with many limitations in terms of travelling, more distance working and less or no socialising activities. Epidemics and pandemics have existed throughout history and impacted societal ways of life and their modes of consumption at different geographical levels (Hays, 2005). Inevitably, each pandemic has influenced the way in which individuals interact, work and live (Snowden, 2020). However, one of the peculiarities of the COVID-19 pandemic is its disruptive impact on the way in which individuals live at both a local and a global level (Salama, 2020). The expansion and spread of pandemics in recent centuries have been aided by large, urban agglomerations (Hang, 2020; Pinheiro & Luís, 2020) and by globalisation and international movement and trade (Bontempi, 2020). In turn, these changes might have triggered a reexamination of individual-level practices in regard to lifestyles and modes of consumption as they relate to the impact on the environment and future generations. These changes have been accompanied by the pervasive use of technologies in people’s everyday life, which in turn might persist even after the pandemic will be over. Even though both positives and negatives have been identified in terms of the environmental impact of the COVID-19 crisis (e.g. an increase in healthcare waste, see Rume & Islam, 2020), the shift to digital forms of learning, consumption and social networking might produce positives due to a routinised (technological) behaviour (Kirby, 2017) that might consciously or unconsciously influence everyday practices even when the pandemic will be over.

Against this background, this paper aims to investigate whether the COVID-19 pandemic has prompted the consideration of new sustainable ways of living.

Therefore, the main research question leading this paper is:

**RQ:** has the advent of the pandemic forced individuals to rethink their lifestyles more sustainably?

To answer this research question and to shed light on individuals’ post-pandemic perceptions of CC, we carried out an empirical investigation on a sample of the UK adult population, during the first wave of the pandemic (June–July 2020), to understand people’s perceptions of CC, while their life was being significantly disrupted by the COVID-19 crisis. The choice of this period is particularly important, as it represents a moment when ‘normality’ was momentarily restored during the summer period and most restrictions were eased. This means that it represented the moment in which people had the opportunity to either return to pre-COVID habits or to adopt new values/behaviours. The originality of this research relies upon the fact that we analysed how the COVID-19 crisis relates to both subjective dispositions towards CC as well as everyday practices relating to CC. Thus, the analysis not only deals with ‘perception’ but also practice, for instance, by exploring both individuals' perception and intention to adopt green/sustainable consumption styles (Stern, 2000) and pro-environmental behaviours (Elliott, 2013).

## Literature Review

Even though there is no scientific evidence to indicate that CC can accelerate COVID-19 direct transmission, the World Health Organization highlights that both global threats are indirectly interconnected, due to the overlapping pressure on health systems (WHO, 2020). However, the question relating to potential interconnections between CC and the transmission of coronavirus is one of the frequent questions that the public asks authoritative figures in relation to COVID-19 (Harvard T.H. Chan, 2020; WHO, 2020). This suggests that public consciousness regarding the potential interconnections between the two phenomena has increased.

COVID-19 has been described as potentially activating collective solidarity, as envisaged by ecological modernisation theorists (Chiles, 2020), as well as increasing the use of technologies and the direct experience of the effects of creating ‘risks without borders’ (Hulme et al., 2020). The COVID-19 crisis has also forced many people on both an individual and collective level to drastically change their lifestyle. This means that the crisis has not only produced consequences from a social, environmental and economic perspective but also has raised questions about the long-term sustainability of consumption-based capitalism in the context of ecological degradation and resource scarcity (Hanafi, 2020). Coronavirus has demonstrated some fragilities of capitalism (Hanafi, 2020) and the necessity to review a model of growth that has led to a serious deterioration of the environment and an international spread of new pandemics. The combination of structural and individual changes that might be observed during crises suggests that breaking the routinised social practice might represent a reflective moment for individuals (Shove, 2004) and a way to voluntarily or involuntarily internalise new (pro-environmental) behaviours. The COVID-19 pandemic established a technological routine due to the imposition of social distancing that might have activated new ways of conceptualising and dealing with individuals’ impact on the environment. Sociological approaches that explore green behaviour have emphasised two main approaches in explaining pro-environmental directions of society by focusing either on individual choices (see Shove, 2010), which have been also explained in terms of social desirability (Elliott, 2013), or structural determinism (see Shove, 2004). However, some intermediate approaches (Shove, 2003) suggest that pro-environmental behaviour might depend more on ‘systemic shifts in routine habits and practices’ (Shove & Warde, 2002, p.13) and context-dependent reflexivity (Pedersen, 2000). In this light, the individual traits of consumers might not be sufficient to explain behaviours that are also highly context dependent (Diamantopoulosa et al., 2003). Interpreting green-oriented solutions as an individual choice, which results from a self-identity (Giddens, 1990) that is free from the structural constraints and class-dependency (Beck, 1992), might not be sufficient to tackle environmental issues. The COVID-crisis might represent an opportunity that shows how the ontological security (Giddens, 1990) is compromised by such an individualistic approach to the environmental problem and reflect on the necessity to combine structural and individual orientations to the problem. Following the Bourdesian approach adopted by Laidley (2013), the combination of structural forces related to class membership and rational choices are associated with different solutions to climate change such as structural solutions to climate change, or more individual market-based and technological approaches.

Green consumption studies define green consumption as associated with consumption practice and lifestyles that are based on or combine environmental, ethical and political values (Barnett et al., 2011). Social practice can include different categories such as work, home and leisure (Barr & Gilg, 2006). However, they can also focus on a specific practice (Lorenzen, 2014), but we argue that scarce attention is given to the potential environmental benefits deriving from an increasing application of Internet technologies in everyday life.

Certain studies have highlighted the capability of generalised crises to trigger prosocial actions, which have limited personal advantages and more social benefits (Wells et al., 2020). However, although the environmental effects of these restrictions have mainly been interpreted as short-term consequences (European Environmental Agency, 2020; Gillingham et al., 2020), the crisis has highlighted human capacity to adapt to global crises and to accept such draconian changes for the collective best interest (Bouman et al., 2021; Herrero & Thornton, 2020). Moreover, several studies have highlighted that social and environmental systems are strongly interconnected in terms of facilitating the spread of new diseases (Dobson et al., 2020). In turn, this might suggest that emphasising the interconnections between the two crises might generate engagement and that COVID-19 might represent a point of shift between the old and new conceptualisations of development.

From a sociological perspective, this critical moment might be interpreted as a ‘socio-cultural shock’ between the sedimented, capitalist habitus of contemporary society and the experience of a new field. At the same time, such a fracture might represent the first step to enhance processes of adaptation and acceptance of new values. In this sense, the Bourdesian concept of habitus helps understand the unconscious ‘chameleon’ (Abrahams & Ingram, 2013) capacity to adapt to change (Bourdieu, 1977, 1990). This means that the ‘dialectical confrontation’ (Bourdieu, 2002, p. 31) between habitus and the fluidity of the contemporary uncertain context might be ‘solved’ by integrating such new values and behaviours in a new, ‘altered’ (Bourdieu & Wacquant, 1992) socio-ecological habitus. Therefore, the literature shows that the ecological or eco-habitus (Eriksen, 2013; Kasper, 2009; Kirby, 2017) depends on both social and ecological practices that develop from a sense of place (Smith, 2001), which in turn might also result in a rejection of the original habitus (Wentworth & Peterson, 2001).

Against this background, this paper explores whether the advent of the pandemic has catalysed UK citizens to rethink their everyday practices, in terms of a more sustainable lifestyle. We investigated these interconnections during the so-called first wave of the pandemic (summer 2020), to capture potential changes in individuals’ perceptions soon after the first spike of the crisis when certain aspects of ‘everyday’ life were restored.

By limiting people’s movement and imposing more restrictions on production activities, COVID-19 has provided an opportunity to rethink societal practices more sustainably. However, as has emerged throughout this review, there is still a need to understand which factors might play a role in influencing the perception of the pandemic as an opportunity to reflect on individual practices in an eco-friendly way. Following certain studies that highlighted how in the UK, age and educational backgrounds are relevant determinants of CC awareness (European Social Survey, 2016), we assume that both age and education play a key role in influencing the perception of COVID-19, as an opportunity to change lifestyles. More specifically, we split this first hypothesis into two sub-hypotheses:

**H1a.** Higher levels of education have a positive impact on the perception of the COVID-19 pandemic, as an opportunity to heighten people’s environmental awareness.

We are assuming that education positively influences the perception of the pandemic as a catalyst to adopt an eco-friendly lifestyle. By contrast, social restrictions have affected older people by increasing the time they spend alone (OfNS, 2020) and by increasing the risk related to mental well-being (Armitage & Nellums, 2020). This suggests that the pandemic might have increased worries for elderly people regarding the disruption of their everyday routine, rather than boosting their environmental orientation. Therefore, we assume that:

**H1b.** The perception of the pandemic as a catalyst for adopting more environmentally sustainable lifestyles varies with age.

Strictly related to social isolation is the use of technologies, to enhance social relationships. Our second hypothesis is related to the diffusion of technologies, accelerated by the restrictions imposed by the pandemic. In fact, as highlighted by this literature review, during the first lockdown, there was an improvement in air quality and a reduction of human-caused noise in several UK cities (UKRI, 2020). In addition to the slowdown of the economy and the reduction of emissions, industrial waste and fossil fuel consumption, certain positive effects on the environment have also been attributed to e-commerce (Siikavirta et al., 2002), the reduction of movement and traveling (Rume & Islam, 2020) and the use of technologies (Elavarasan & Pugazhendhi, 2020). Therefore, H2 hypothesises the following:

**H2.** The coronavirus pandemic made users realise that digital skills are important in reducing their impact on the environment.

The relationship between the possessions of digital competencies and environmental participation has been scarcely considered by the literature. Extensive attention has been devoted to studying media products (mainly news media) as a predictor of environmental behaviour (see e.g. Östman, 2014) and awareness (Arlt et al., 2011). Zhang and Skoric (2018) analysed the role of media consumption in shaping citizens’ environmental behaviours finding that social media might encourage environmental consumerism among nonmembers of Environmental Non-Governmental Organisations. Shah et al, (2021) suggest that exposure to climate change-related information on social media positively influences users’ pro-environmental behaviours. However, this literature focuses on the exposure to media content rather than on the concrete use of Internet technologies to limit the physical impact that individuals have on the environment.

Finally, the optimistic interpretation of the current socio-ecological reality suggests the possibility of creating a new global culture, based on the values and behaviours that are oriented towards sustainable principles (Galvani et al., 2020). In this sense, the global crisis might represent a ‘dialectical confrontation’ for people between their ‘old’ and ‘new’ lifestyles, with a potential alteration of the eco-habitus (Bourdieu, 1977; 1990; Bourdieu & Wacquant, 1992). Therefore, the third hypothesis is directly connected to this opportunity:

**H3.** Citizens are confident that their digital skills will reduce the impact of their lifestyle on the environment, even after the coronavirus crisis is over.

In addition to testing H1–H3, we are interested in investigating the degree of polarisation in the opinions of the UK population concerning: (i) the causes of CC (i.e. the natural origin of CC vis à vis the anthropogenic derivation of the phenomenon); (ii) the possible link between pollution and the severity of the symptoms of COVID-19; (iii) the perception of the COVID-19 pandemic as an opportunity to reset societal lifestyles and embrace a more eco-friendly way of life; (iv) the importance of digital skills in reducing environmental impact; and (v) the intention to continue to use digital skills after the COVID-19 pandemic, to reduce the environmental impact.

Polarisation is a concept formally introduced in the econometric literature by Esteban and Ray (1994) and is based on the notion of identification–alienation. More specifically, polarisation refers to the creation of groups within a society, with a high degree of intra-group homogeneity (identification) and strong inter-group heterogeneity (alienation). In a polarised society, each individual identifies themself with a particular group and feels alienated from other groups. Even though the seminal contribution of Esteban and Ray (1994) was aimed at proposing a measure of polarisation in income distribution, subsequent literature has proposed various measures of social polarisation. According to Fusco and Silber, social polarisation refers to ‘the measurement of the distance between different social groups defined on the basis of a variable such as race, religion, or ethnicity’ (2014, p. 844). In our particular case, we believe that educational levels may be an important driver of polarisation with regard to the aforementioned issues. Therefore, another explorative aim of the paper is to assess the degree of polarisation in relation to issues i, ii, iii, iv and v among various social groups, defined by educational attainment.

## Methodology

### Sample

This work uses an online sample of the UK adult population (1013 respondents), collected during the pandemic (June–July 2020), that included a mixture of educational backgrounds (Table 1 panel A), age groups (Table 2) and different annual household incomes (see Table 1 panel B r). In Table 1A panel B (in the appendix), we report descriptive statistics for other socio-demographic variables (gender composition of the sample, area of residence and employment status). We did not adjust the sample for the digitally excluded population because we aimed to focus on differences among internet users. The sample appropriately captures the demographic stratification of the UK population, but it was a quota sample based on the voluntary participation of respondents. One drawback of using Internet panels is the voluntary nature of recruiting, which does not allow to calculate sampling error. Hence, even though for the sake of simplicity, in our comment of the results we will refer to the UK population, we are careful in extending the results outside the sample that we have studied.

**Table 1.** Descriptive Statistics for Education and Income levels.

A. Education qualifications.			
	Frequency	Percentage	Cumulative percent
Did not complete high school, no diploma	90	8.9	8.9
High school graduate	269	26.6	35.4
Some college credits, no degree	219	21.6	57.1
Bachelor’s degree	327	32.3	89.3
Master’s degree	84	8.3	97.6
Doctorate	24	2.4	100
Total	1013	100	
B. Income Levels.			
	Frequency	Percentage	Cumulative percent
Under £10k	76	7.97	10.91
>10,000£–≤25,000£	281	29.49	40.4
>26,000£–≤50,000£	397	41.66	82.06
>50,000£–≤100,000	171	17.94	100
>100,000£	28	2.94	2.94

**Table 2.** Age of Respondents.

	Age			Total
	18–34	35–54	55+	
Total	271	367	375	1013

**Table 1A.** Frequency Distribution of Variables y^0^, y^1^, y^2^, y^3^ and y^4^.

	y^0^		y^1^		y^2^		y^3^		y^4^	
	Freq	Freq %	Freq	Freq %	Freq	Freq %	Freq	% Freq	Freq	% Freq
1	150	15.74	53	5.56	43	4.51	69	7.24	69	7.24
2	259	27.18	104	10.91	72	7.56	119	12.49	138	14.48
3	250	26.23	225	23.61	229	24.03	342	35.89	329	34.52
4	189	19.83	419	43.97	438	45.96	334	35.05	322	33.79
5	105	11.02	152	15.95	171	17.94	89	9.34	95	9.97

### Method

The hypotheses were explored by identifying diverse groups, characterised by specific socio-demographic traits in relation to their CC awareness and their intention to change their lifestyle (also thanks to their digital skills) even after the end of the crisis. Our analysis also looked at the heterogeneity of the groups, to explore whether there was polarisation both between and within the groups.

To calculate the level of polarisation, we adopted the Leti index. We will, firstly, describe our proposed indicator of polarisation (R1-R5). Indeed, one of the steps for the construction of the indicator is also useful for testing our hypotheses H1, H2 and H3. Consider a generic ordinal variable, y_i_, with I categories. The Leti index is a measure of heterogeneity for an ordinal variable, proposed by Leti (1983) and could be calculated as follows

(1) $$L=2\overset{I-1}{\underset{i=1}{\sum }}F\left(y_{i}\right)\left(1-F\left(y_{i}\right)\right)$$

where F (y_i_) is the cumulative relative frequency of the ordinal, y_i_.

The Leti index equals 0 if the frequencies are concentrated in one category (homogeneity), while it is equal to (I−1)/2 * (1–1/n^2^) if heterogeneity is highest; *n* indicates the size of our sample or the population size if we have census data (in the case of an even number of categories, the second term of the product is equal to 1). Heterogeneity reaches its maximum when frequencies are equally split between category 1 and the highest category I. The Leti Index allows measuring individual heterogeneity in an ordinal variable without neglecting the qualitative nature of the (not numeric) variable. If the standard deviation is calculated, the implicit assumption is that we are considering a qualitative ordinal variable as if it were a purely quantitative one (as, for instance, income). Polarisation implies the creation of groups that are homogeneous within them and heterogeneous with each other. In this context, the Leti Index is a measure of total heterogeneity in our sample; thus, we cannot directly use it to measure polarisation. However, Grilli and Rampichini (2002) have shown that the Leti index can be broken down (as in the case of the variance) into two components: the within-group heterogeneity and the between-group heterogeneity. For instance, suppose that a population of size *n* can be divided into j groups (j = 1,…,J), we will indicate with n_i,j_ the frequency observed for category i in group j, while n_j_ indicates the size of group j. The within-group heterogeneity can be calculated as follows

(2) $$L^{W}=\overset{J}{\underset{j=1}{\sum }}\frac{n_{j}}{n}L_{j}$$

where L_j_ is the Leti index, calculated inside each group j. Therefore, L^w^ is a measure of how much the components of the J groups are dispersed inside each group.

The between-group heterogeneity is instead given by

(3) $$L^{B}=2\overset{J}{\underset{j=1}{\sum }}\frac{n_{j}}{n}\overset{I-1}{\underset{i=1}{\sum }}F\left(y_{ij}\right)\left[F\left(y_{ij}\right)-F\left(y_{i}\right)\right]$$

where F (y_ij_) is the cumulative relative frequency of y_i_ in group j. Thus, L^B^ is a measure of how much a group is different from another group.

Subsequently, a simple measure of polarisation is given by (see also Mussini, 2018)

(4) $$P^{M}=\frac{L^{B}}{1+L^{W}}$$

The index of polarisation proposed is equal to 0, when all the groups have the same relative frequency distribution, in other words, when there is not ‘between-groups heterogeneity’. The index goes up as between groups increase. At the same time, P^M^ decreases as L^W^ increases, since an increase in within-group heterogeneity also implies that people have more difficulty in recognising themselves as part of a particular group. The number 1 is added in the denominator of equation (4), to avoid positive divergence of the indicator when the within-group heterogeneity is 0. Note also, that when L^w^ = 0, L = P^M^ = L^B^. Hence, this allows the maximum for P^M^ to be defined as the maximum value that L can assume.^1^

It is perfectly possible to compare the level of polarisation on a topic captured by an ordinal variable, y_i_, with another, associated with a different variable, x_i_, conditioned by the fact that I is equal for both variables. We are interested in evaluating the level of polarisation regarding both the scepticism towards CC and the perceived impact of the COVID-19 pandemic on various aspects of the CC issue: the adoption of eco-friendly behaviours, the role of digital skills in reducing the environmental impact and the willingness to continue to use and improve digital competencies after the pandemic, to increase sustainability. Table 3 presents the survey questions that allow us to evaluate these perceptions.

**Table 3.** Questions Related to the Perception of CC and Intentions.

Q22. Please indicate to what extent you agree or disagree with the following statements about CC by ticking one box in each row: CC is just a natural fluctuation in the Earth’s temperature (variable coded as y0)				
1. Strongly disagree	2. Disagree	3. Neither agree nor disagree	4. Agree	5. Strongly agree
Q26. To what extent do you agree or disagree with the following statement				
1. Strongly disagree	2. Disagree	3. Neither agree nor disagree	4. Agree	5. Strongly agree
CC will make the COVID-19 pandemic worse? Variable coded as y1				
We can use coronavirus to reset our lives with regard to sustainability. Variable coded as y2				
I will use my digital skills to reduce the impact of my lifestyle on the environment even after the coronavirus crisis is over. Variable coded as y3				
The coronavirus pandemic made me realise that digital skills are vital in reducing my carbon footprint. Variable coded as y4				

In Table 2A (panel A) in the appendix, we present the relative frequencies of each possible answer for these variables. Our group partition is based on the level of education. If we calculate the polarisation on the topic reported in Table 3 among these groups, by applying equation (4) to the raw data, our results may be influenced by other population compositional effects (for instance, employment status, level of income, etc.). Thus, we need to consider other confounding effects to be sure that we are capturing polarisation among the j groups of interest.

**Table 2A.** Other socio-Demographic Variables.

Gender	in %	UK official mid 2019 population
Male	47.63	49.04
Female	52.37	50.86
Area of residence^a^	In %	
South	45.69	44.91
Midlands and Wales	20.91	20.84
North and Scotland	30.78	31.4
Northern Ireland	2.61	2.83
Employment status		
Employee (Full + part time workers)	56.24	
Self-employed	4.83	
Student	4.09	
Unemployed	6.19	
Retired	17.21	
Other non-professional conditions	11.44	
Coming from urban/rural area		
Rural area^b^	18.26	
Urban area	81.74	

^a^South includes the following regions: London, South-East, South-West, East North and Scotland includes: Yorkshire and the Humber, North East, North West, Scotland Midlands and Wales includes: West Midlands, East Midlands and Wales

^b^last official statistics about rural/urban population are for 2018 and indicate that the 17% of the UK population lives in rural areas

To isolate the polarisation across educational levels, we ran five, ordered, logit regressions, in which the dependent variables were alternatively the variables reported in Table 4*,* while the r.h.s variables were the age of the respondents, the level of education and the level of income, with a dummy for the place of residence. After estimating our models, we only kept the variable capturing the level of education; this was allowed to vary among individuals, while all other controls were fixed to the same value for every i.

Then, we predicted the probability of being in each separate category of each of our dependent variables at the individual level. Finally, we assigned each individual to the category for which the probability was higher, by obtaining a new variable that we called *bw_y*^*k*^*, with k = 0,1,2,3,4*. This new variable was free from individual heterogeneity (since we assigned the same characteristics to all the individuals). Thus, this variable was used to calculate L^B^, as reported in equation (3). Note that these regressions are not only useful for calculating the between-group heterogeneity, but they are also helpful in testing the aforementioned H1–H3.

To obtain a measure of within-group heterogeneity, we ran a separate, ordered, logit model for each level of education. Therefore, for each individual, i, belonging to education level, j, (with j = less than a high school certificate, high school certificate, bachelor’s degree or higher than a bachelor’s degree), we predicted the probability of being in each one of the *y*^*k*^ categories. Then, we assigned each individual to the category for which the probability was the highest, obtaining, therefore, a new variable that we called *wi_y*^*k*^. The latter variable was used to calculate L^w^ as reported in equation (2). Finally, the indicator of polarisation among the educational levels of each individual was calculated, according to equation (4).

## Results

Table 4 presents the results of the five multivariate, ordinal, logit regressions that we ran using the dependent variables, described in Table 4. The names of the regressors are self-explanatory. In Table 3A (in the appendix), we report the same results obtained running a more complex model where in addition to the age of the respondent we have included also its square have to test the potential non-linear relationship between ageing and attitudes towards CC^2^. Furthermore, the models estimated in Table 3A also include a more detailed income/employment status categorisation, a dummy for the area of residence (Northern Ireland, North, South, Midlands and Wales). The results do not change a lot with respect to those reported in Table 4.

**Table 4.** Multivariate Ordinal Logit Regressions.

	(1) y^0^	(2) y^1^	(3) y^2^	(4) y^3^	(5) y^4^
Age	−0.011 (0.005)**	−0.012 (0.005)**	0.001 (0.005)	−0.015 (0.005)***	−0.009 (0.005)*
Gender= female	0.057 (0.123)	0.119 (0.125)	0.258 (0.127)**	0.141 (0.122)	0.205 (0.122)*
Education					
Did not finish h. school	0.576 (0.222)***	0.255 (0.244)	0.157 (0.258)	−0.140 (0.236)	−0.204 (0.235)
High school	Ref	Ref	Ref	Ref	Ref
Bachelor	−0.168 (0.131)	0.381 (0.134)***	0.283 (0.138)**	0.529 (0.138)***	0.324 (0.136)**
MSc_PhD	0.061 (0.249)	0.672 (0.241)***	0.644 (0.213)	0.800 (0.225)***	0.528 (0.213)**
Income					
≤10,000£	0.145 (0.229)	−0.441 (0.259)*	−0.183 (0.281)	0.141 (0.263)	−0.606 (0.264)**
>10,000£–≤25,000£	0.017 (0.142)	−0.063 (0.148)	−0.173 (0.143)	0.130 (0.141)	−0.188 (0.140)
>25,000£–≤ 50,000£	Ref	Ref	Ref	Ref	Ref
>50,000£–≤ 100,000£	0.081 (0.169)	−0.157 (0.175)	0.021 (0.170)	0.100 (0.167)	0.212 (0.172)
>100,000£	0.228 (0.430)	−0.893 (0.405)**	−0.496 (0.426)	−0.075 (0.424)	−1.384 (0.466)***
Employment status					
Employee	Ref	Ref	Ref	Ref	Ref
Self-employed	−0.643 (0.270)**	0.031 (0.249)	0.185 (0.252)	−0.103 (0.277)	−0.065 (0.275)
Student	−0.729 (0.325)**	0.259 (0.358)	0.244 (0.295)	−0.128 (0.278)	−0.144 (0.242)
Unemployed	−0.455 (0.232)**	0.314 (0.277)	0.258 (0.267)	−0.127 (0.262)	−0.171 (0.257)
Retired	−0.052 (0.203)	0.024 (0.199)	−0.325 (0.209)	0.089 (0.211)	−0.258 (0.200)
Other	0.095 (0.195)	−0.106 (0.198)	−0.203 (0.233)	−0.699 (0.191)***	−0.513 (0.199)***
Coming from rural area=Yes	0.168 (0.168)	0.126 (0.166)	−0.110 (0.170)	0.129 (0.167)	−0.046 (0.166)
*N*	953	953	953	953	953

Certain observations were excluded, as information relating to income was missing.

Heteroskedasticity Robust Standard errors in parentheses; cutoff parameter not reported to save space.

*p < 0.10, ** *p* < 0.05, *** *p* < 0.01.

**Table 3A.** Multivariate ordinal logit regressions.

	(1)	(2)	(3)	(4)	(5)
	y^0^	y^1^	y^2^	y^3^	y^4^
Age	−0.010 (0.005)*	−0.013 (0.006)**	−0.000 (0.006)	−0.016 (0.005)***	−0.008 (0.005)*
Age^2^	−0.000 (0.000)	−0.000 (0.000)	−0.000 (0.000)	0.000 (0.000)	−0.000 (0.000)
Gender=Female	0.059 (0.124)	0.136 (0.126)	0.261 (0.129)**	0.106 (0.125)	0.198 (0.126)
Education					
Did not finish high school	0.651 (0.231)***	0.244 (0.257)	0.180 (0.272)	−0.168 (0.243)	−0.197 (0.242)
High school	Ref	Ref	Ref	Ref	Ref
Bachelor’s degree	−0.166 (0.132)	0.371 (0.136)***	0.247 (0.141)*	0.522 (0.142)***	0.301 (0.140)**
MSc_PhD	0.009 (0.255)	0.698 (0.245)***	0.663 (0.216)***	0.803 (0.234)***	0.454 (0.219)**
Annual income in £					
<10,000	−0.210 (0.273)	0.176 (0.282)	0.213 (0.321)	−0.011 (0.311)	−0.131 (0.324)
≥10,000–< 15,000	0.040 (0.227)	0.213 (0.268)	0.074 (0.243)	0.322 (0.226)	0.211 (0.229)
≥15,000–< 20,000	−0.159 (0.228)	0.447 (0.263)*	0.143 (0.243)	−0.041 (0.230)	−0.041 (0.229)
≥20,000–< 30,000	Ref	Ref	Ref	Ref	Ref
≥30,000–< 40,000	−0.181 (0.198)	0.280 (0.188)	0.167 (0.196)	−0.023 (0.201)	0.165 (0.199)
≥40,000–< 50,000	−0.159 (0.232)	0.377 (0.227)*	0.609 (0.232)***	0.020 (0.207)	0.270 (0.214)
≥50,000–< 60,000	−0.235 (0.261)	0.437 (0.283)	0.272 (0.260)	0.521 (0.275)*	0.506(0.272)*
≥60,000–< 70,000	−0.384 (0.229)*	0.151 (0.256)	0.439 (0.251)*	−0.075 (0.256)	0.258 (0.237)
≥70,000–< 100,000	0.157 (0.264)	0.343 (0.250)	0.401 (0.246)	0.161 (0.252)	0.475* (0.261)
≥100,000	0.156 (0.396)	−0.650 (0.367)*	−0.358 (0.396)	−0.088 (0.335)	−0.372 (0.430)
Employment status					
Other	−0.179 (0.980)	−0.764 (0.903)	0.032 (1.180)	−0.376 (0.985)	−1.905 (0.657)***
Full-time dep. worker (≥30 hours)	Ref	Ref	Ref	Ref	Ref
Part time dep. worker (<30 hours)	0.030 (0.184)	−0.159 (0.208)	0.035 (0.206)	0.060 (0.203)	0.023 (0.181)
Looking after the home	0.079 (0.244)	−0.090 (0.251)	−0.278 (0.270)	−0.426 (0.244)*	−0.281 (0.268)
Long-term sick/disabled	0.243 (0.321)	−0.425 (0.324)	−0.133 (0.399)	−1.091 (0.294)***	−0.759 (0.305)**
Self-employed	−0.606 (0.272)**	−0.036 (0.267)	0.162 (0.261)	−0.076 (0.295)	−0.113 (0.301)
Student	−0.698 (0.368)*	0.180 (0.397)	0.233 (0.326)	−0.171 (0.296)	−0.194 (0.287)
Unemployed	−0.375 (0.235)	0.172 (0.270)	0.259 (0.275)	−0.066 (0.274)	−0.266 (0.268)
Retired	−0.049 (0.252)	0.011 (0.235)	−0.245 (0.251)	0.072 (0.251)	−0.209 (0.236)
Place of residence					
Midlands and Wales	Ref	Ref	Ref	Ref	Ref
North and Scotland	−0.000 (0.162)	0.130 (0.174)	0.089 (0.172)	0.102 (0.172)	−0.027 (0.165)
Northern Ireland	0.203 (0.349)	−0.023 (0.370)	0.285 (0.398)	−0.042 (0.341)	−0.089 (0.388)
South	−0.013 (0.153)	−0.075 (0.164)	0.087 (0.159)	0.035 (0.161)	0.098 (0.163)
Urban or rural context					
Rural areas	0.344 (0.184)*	0.070 (0.183)	−0.154 (0.188)	0.099 (0.182)	0.000 (0.183)
Small towns	Ref	Ref	Ref	Ref	Ref
Urban areas	0.304 (0.132)**	−0.120 (0.136)	−0.047 (0.137)	−0.030 (0.135)	0.190 (0.137)
*N*	953	953	953	953	953

Notes: Certain observations were excluded, as information relating to income was missing. Heteroskedasticity Robust Standard errors in parentheses; cutoff parameter not reported to save space.

*p < 0.10, ** *p* < 0.05, *** *p* < 0.01.

Note that Table 4 does not coincide with the sample size, due to missing answers in relation to the dependent variables or on account of certain regressors (especially income).

Interestingly, only age and education had a statistically significant effect in almost all the columns. The results indicated that, on the one hand, older people are more aware that CC is related to human activities^3^; however, on the other hand, they are less prone to believing that digital competencies could also be helpful post-pandemic, in reducing environmental impacts (columns 4 and 5). The regression also showed a negative relationship between age and the belief that the COVID-19 pandemic may be worsened by CC.

Considering education, those who had not completed high school were most sceptical regarding the anthropogenic origin of CC. In fact, keeping all the other variables at their mean level, an individual with less than a high school diploma has a higher probability of % being sceptical than those in the reference category (17% against the 10% estimated for the reference). In line with these findings, we have established that most educated people are also those who believe that they can use their digital skills to reduce their environmental impact and that the COVID-19 pandemic might also represent an opportunity to adapt our lifestyle to a more sustainable one. More educated people also linked COVID-19 and pollution and tended to agree that CC might exacerbate the negative effects of the pandemic. Note that education is the only variable that is always significant in all columns. The results, associated with other variables (income, gender, employment status, place of residence and size of city), are unstable in terms of statistical significance across the columns. Therefore, this result further justifies the choice of focusing on education as the main source of polarisation among social groups.

**Figure 1.**
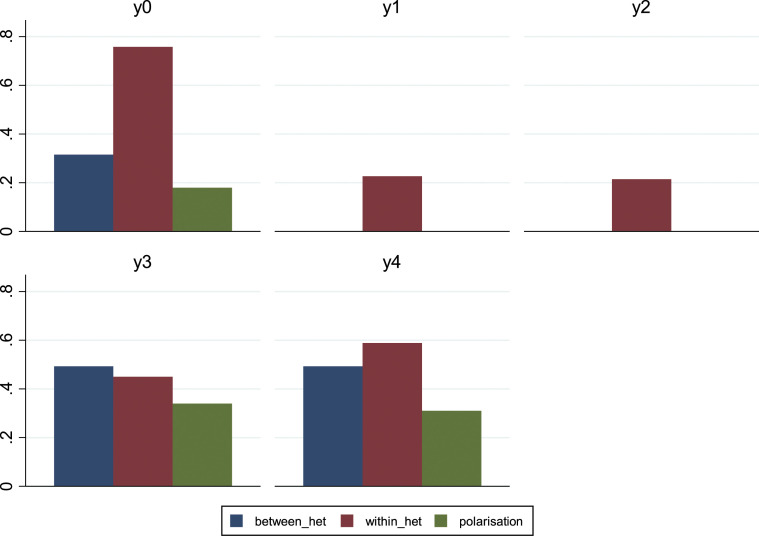
Polarisation within educational groups.

Interestingly, when we predicted the category for variable y^0^ to proceed to the calculation of the numerator in equation (4), we observed a perfect separation between individuals with a low level of education (i.e. those with less than a high school certificate) and those having at least a high school certificate, with the responses of the former concentrated in the category, ‘Agree’, and the responses of the latter in the category, ‘Disagree’ (see Table 5).^4^ Despite this finding, the high degree of heterogeneity within each group implies that polarisation is particularly low (the indicator is near to its minimum). In figure 1 we report the between-groups heterogeneity, the within-groups heterogeneity and the polarisation index for each dependent variable. Hence, although lower educated people are more likely to believe that CC is a natural phenomenon, this does not automatically mean that each individual with a low/high standard of education has the same opinion as other people, who hold the same qualification. Between-group heterogeneity allows for the comparison of individuals that differ from one another only in terms of their educational level. However, polarisation also requires a strong group identity, that is, in our specific case, a high degree of homogeneity within each educational level. This is not the case for the UK, at least according to our results.

**Table 5.** Predicted Category by Educational Level, Fixing all Other Characteristics.

	CC is just a natural fluctuation in the Earth’s temperatures				
	Less than h. School	H. School	Bachelor	MSc/PhD	Total
Strongly agree	0	0	0	0	0
Agree	0	461	303	124	888
Neither agree nor disagree	0	0	0	0	0
Disagree	84	0	0	0	84
Strongly disagree	0	0	0	0	0

Considering variables y^1^ and y^2^, we maintain that the indicator of polarisation is equal to 0, given that other characteristics are fixed, and the level of education predicts the same category for all groups, leading then to L^B^ = 0. We also report that for both these variables, the within-group heterogeneity is lower than that calculated for variable y^1^, indicating therefore, that inside each educational level, these issues are less controversial than CC. Concerning y^3^ and y^4^, we maintain that by fixing all other characteristics, education perfectly separates those with a high school certificate (or less) from those who have at least a bachelor’s degree, with the responses of the former ending up in the category, ‘Undecided (Neither agree nor disagree)’, and the latter in the category, ‘Agree’. This implies the same degree of between-group heterogeneity for both variables. Note that the L^B^ for these two variables is higher than that calculated for y^1^. The higher heterogeneity is mainly driven by the fact that in the case of the question regarding CC, only those with less than a high school diploma were classified in a different category from the rest of the sample, while in the case of y^3^ and y^4^, the sample is almost split exactly into two halves: around 56% of the sample used in the regressions (i.e. those individuals with a high school certificate or less) was composed of individuals, who were undecided about the proposed topic, while the remaining observations were all classified in the category, ‘Agree’. Confronting the index of polarisation, we report that this value is higher for variable y^3^ than for y^4^ (0.34 vs. 0.31), and this result is exclusively driven by the lower, within-group heterogeneity for variable y^3^. In other words, individuals with the same level of education tend to have a more similar opinion on this specific topic than that captured by variable y^4^. Note that in our case 0≤ P^M^≤1.99, the level of polarisation among educational groups in the UK regarding all these topics does not seem to be particularly accentuated.

## Discussion

The results of the ordinal regression models do not support H1b, given the non-statistical influence of age, in relation to viewing the pandemic as an opportunity to reset societal lifestyles. However, on further examination of the factors playing a role in influencing the perception of the pandemic, as an opportunity to reflect on individual practices in an eco-friendly way, the second hypothesis explored whether the COVID-19 pandemic made users realise that digital skills are important in reducing their impact on the environment. The regression models show that older people are more aware that CC is related to human activities, but are less inclined to believe that an increase in the use of digital technologies for everyday activities could be beneficial to the environment in the post-pandemic. However, this might be explained by the fact that older individuals tend to have fewer digital competencies (Niehaves & Plattfaut, 2013; Ragnedda et al., 2019). In turn, this might have increased the challenges of adapting to technologies during the pandemic and affected their confidence in their ability to minimise their environmental impact by using digital technologies. Moreover, during the first wave of the pandemic, younger people (aged 16 to 29 years) were more likely than older respondents to use different technologies as a means of coping with the restrictions. Another possible explanation might be that older individuals are aware of the issues of CC, but they also have a shorter time horizon than younger people, and they may be prone to believe that younger generations will have to deal with CC. Various scientific research studies have studied the relationship between COVID-19 diffusion/severity of symptoms and air pollution (Comunian et al., 2020), but there is insufficient scientific evidence of a connection between air pollution and COVID-19 diffusion (CEBM, 2020) and no evidence of a connection between CC and COVID-19 (Harvard T.H. Chan, 2020; WHO, 2020). However, the findings that highlight a potential correlation between higher levels of pollution and higher COVID-19 mortality have received a great deal of attention from traditional media, for example, the BBC^5^. Hence, it is quite surprising that the most frequent users of these sources of information are also less likely to agree with the statement captured by variable y^1^. It should be said that even though older individuals are more aware of the anthropogenic origin of CC, and thus of the link between pollution and CC, they may also be convinced that CC is a future-oriented phenomenon (Ruiu et al., 2020) by comparison with COVID-19, and this might cause them not to identify a link between the two. Furthermore, this misperception may be exacerbated by the fact that – *ceteris paribus* – an older individual is at greater risk of death than a younger individual, should he/she contract the virus. Therefore, COVID-19 might be perceived as an immediate risk for an older individual, whereas CC is considered a risk for future generations.

Considering H1a, those with a lower level of education also seem more sceptical regarding the anthropogenic origin of CC. By contrast, higher levels of education are associated with the perception that digital skills can be used to reduce environmental impact. Those with a higher level of education also tend to perceive the COVID-19 pandemic as an opportunity to adapt their lifestyles to become more sustainable.

Finally, more educated people link COVID-19 and pollution and tend to agree that CC may worsen the consequences of the virus. Given that results regarding education were significant across all models, we focused on education as a potential source of polarisation among social groups. A stronger in-group homogeneity was found between people with the same level of education and the perception that digital skills would have a positive effect on the environment, even in the post-COVID-19 era. This result is reasonable, given the existing digital divide between those with a higher level of education and those with a lower level of education, both in terms of access to digital resources and their online activity profiles (Blank & Groselj, 2014; Ragnedda, 2017, 2020). During the pandemic, the former may have increased their awareness of the importance of distance working in terms of reducing the environmental impact of traditional systems of work organisation, whereas the latter, being less involved in distance working activities, tended to have limited opportunities to reflect on this point and, therefore, less clear opinion with regard to this issue.

The potential alteration of the eco-habitus (Bourdieu, 1977; 1990; Bourdieu & Wacquant, 1992) also heightens awareness that digital skills might help increase environmental sensitivity, by using technologies to reduce the impact of people’s lifestyle on the environment, even after the end of the COVID-19 crisis. A combination of structural and individual changes might be needed to understand how individual needs are constructed and reproduced. Shove (2004) highlighted that the techno-optimistic approach has failed to consider individual ordinary practice, by contrast focusing on a deterministic approach based on the simple promotion of certain goods. On the other hand, approaches based on an ABC paradigm (attitude-behaviour-choice) have disproportionally emphasised individual choices as a trigger for pro-environmental behaviour (Shove, 2010). However, such an individualised approach to consumption, which gives disproportionate attention to the empowerment of users in driving the market and policies, should also consider the structural factors that create opportunities. In other words, ‘breaking old habits’ (Stern, 2000) also involves the context that establishes the rules and favours the emergence of new practice (Shove, 2010). This means that the ‘dialectical confrontation’ (Bourdieu, 2002) between habitus and context might activate the development of a new ‘altered’ (Bourdieu & Wacquant, 1992) socio-ecological habitus. Breaking the routinised social practice with a new practice might also represent a reflective moment, which in turn interacts with personal predispositions and behaviours. In the context of the COVID-19 pandemic, the contextual restrictions broke the social routine through a ‘socio-cultural shock’ between the sedimented social practice and a new technological practice, which in turn might become a new routine. Moreover, this breaking point might represent a reflective opportunity for people to develop a capacity to adapt to change (Bourdieu, 1977, 1990) through a new understanding of how they can reduce their environmental impact without excessive discomfort. In fact, we referred to ‘opportunities’ created from the combination of structural (e.g. fewer opportunities to work from home, rules and existing individuals’ background) and individual traits (predisposition towards environment and technologies) that might make individuals realise that maintaining some technological components in their everyday life might not affect their comfort while benefitting the environment. In turn, the routinised pro-environmental attitudes and behaviours might represent the favourable conditions for the institutional frame to re-direct the techno-orientation of society towards pro-environmental practice even after the COVID-19-crisis will be over.

Finally, the analyses suggest that education is an important factor in raising people’s awareness of the anthropogenic nature of CC and in allowing a better understanding of the importance of digital skills in reducing their impact on the environment. However, when we used educational levels to calculate social polarisation regarding these topics, the population of the UK is barely polarised. In particular, we found that although alienation existed between groups in relation to certain investigated topics (i.e. heterogeneity in the opinions expressed by different groups), the identification was not significant, leading to a low level of polarisation. Therefore, on the one hand, we found that education spurs CC awareness in addition to being positively correlated to the belief that COVID-19 may also represent an occasion to rethink our lifestyle; on the other, educational levels do not polarise society in two opposite and hardly conciliable positions. This makes education an extremely useful policy instrument also in the ambit of an eventual strategy to mitigate CC.

## Conclusions and Limitations

This paper suggested that higher educated people show increased levels of awareness in terms of reducing their environmental impact. We have speculated that higher educated workers might have experienced distance working as a way to reduce their environmental impact due to work organisation, which might also be true for using technologies for additional activities such as consumption behaviours and for limiting their travelling and moving. By contrast, less educated people might not have had the opportunity to work from home and reduce their movements, thus having a less clear opinion with regard to this issue. This represents a useful insight for both the UK ‘green recovery’ from COVID-19 to achieve the net-zero emissions target by 2050 and the European Green Deal plan that also focuses on coupling digital and green transformation to achieve no net emissions of greenhouse gases in 2050 (European Commission, 2019). This suggests that the structural conditions that are external and not dependent on individual choices are fundamental to increase at least the opportunities for people to explore the technological alternative for working, consuming and limiting their movements. However, the contextual conditions (e.g. related to access and possibility of acquiring digital competencies) should be integral to such a twin approach. In this direction, it will be pivotal to further study the relationship between individual choices and structural opportunities, by also exploring specific types of consumer behaviours, work preferences (where possible) and people movements/travelling.

This study focused on some specific variables that might play a role in influencing the pro-environmental use of technologies to reduce individuals’ impact on the environment in the post-pandemic. However, this study is only explorative and introduces several new questions that should be further explored by future research. The generalisation of the considerations should be taken with caution given that the sample is not probabilistic, and respondents voluntarily accepted to complete the questionnaire. For example, we used some demographic variables, such as income, gender, employment status, place of residence, size of the city, education and age, to predict the perception of the COVID-19 pandemic as an opportunity to adapt to green lifestyles and consumption. However, the effects of education and age alone might not be the only factor in predicting future pro-environmental engagement. On the other hand, the literature shows that age and education are good predictors of the use of technologies. Since this study focused on the exploration of the relation between the use of digital technologies and intention to engage with green choice, these variables were considered central to the scope of this paper. However, further research should also consider the mediated effects of personal preferences and psychological traits. Moreover, this study can only offer a general overview of how the awareness that digital competencies can reduce the individual impact on the environment. However, this needs to be further explored in relation to different types of activities and consumption behaviours. Finally, this is a cross-sectional study that does not allow for comparison with previous circumstances and make it not possible to consider potential effects of the technological acceleration compared to previous positions.

## Notes

1 In our specific case, if L^w^=0, the maximum value of our indicator is [((5–1)/2)*(1–(1/(1013 ^ 2))]=1.99.

2 To avoid any issues relating to a strong correlation between age and age^2^, we demeaned the variable age.

3 Keeping all other variables at their mean level, the estimated marginal effect suggests that an increase of 1 in age leads to an increase of 0.1% in terms of the probability of strongly disagreeing with the statements reported in variable y^0^.

4 The polarisation indicator is calculated using the prediction from the most complete model (that reported in Table 3A in the appendix).

5 See, for instance, https://www.youtube.com/watch?v=RSZoDTvgEr8.
